# Late-Onset Proximal Myotonic Myopathy (PROMM): A Rare Presentation in an Adult

**DOI:** 10.7759/cureus.50711

**Published:** 2023-12-18

**Authors:** Vinit Deolikar, Keyur Saboo, Sunil Kumar, Sourya Acharya, Sonali Chavan

**Affiliations:** 1 Department of Medicine, Jawaharlal Nehru Medical College, Datta Meghe Institute of Higher Education and Research, Wardha, IND; 2 Department of Medicine, Datta Meghe Medical College, Datta Meghe Institute of Higher Education and Research, Nagpur, IND

**Keywords:** steinert’s disease, autosomal dominant, dominant multisystem myopathy, proximal myotonic myopathy, myotonic dystrophy

## Abstract

Proximal myotonic myopathy (PROMM) is normally associated with bilateral proximal weakness of lower limbs, slight elevation of liver enzymes, and cataracts. Myotonic dystrophy and PROMM are both autosomal dominant disorders, but gene study is completely normal in the case of PROMM. The most important differential diagnosis of PROMM is myotonic dystrophy. In our case, we reported late-onset PROMM in a patient 42 years old whose symptoms started at the age of 33 years; genetic evaluation of both myotonic dystrophy type 1 and myotonic dystrophy type 2 came out to be normal; therefore, the diagnosis of exclusion PROMM was made, which is a rare entity.

## Introduction

Recently, there has been a newly defined group called dominant multisystem myopathies (DOMMOP); both myotonic dystrophy and proximal myotonic myopathy (PROMM) are covered under this group. Proximal myotonic myopathy (PROMM) is autosomal dominantly inherited and presents as myotonia, proximal muscle weakness, muscle pain, and cataracts. Both myotonic dystrophy and PROMM have the same clinical features, but the genetic basis of both disorders is different [1].

Myotonic dystrophy type 1 or Steinert’s disease is caused due to the repeated expansion of trinucleotide cytosine-thymine-guanine (CTG) in the dystrophia myotonica protein kinase (DMPK) gene, whereas cytosine-thymine-guanine (CTG) expansion in the cellular nucleic acid-binding protein (CNBP) gene leads to type 2 myotonic dystrophy [2]. In contrast, the gene locus for PROMM has not been visualized. PROMM has a more benign course as compared to myotonic dystrophy.

The patient with PROMM generally has no symptoms of brain involvement such as mental retardation and hypersomnia, which are more commonly seen with myotonic dystrophy type 1. As the chromosomal location of the PROMM is still not known, the diagnostic test is not available [3,4].

## Case presentation

A 42-year-old male presented to the outpatient department of medicine with complaints of bilateral lower limb weakness and difficulty in walking for eight to nine years; the patient also complained of easy fatiguability for eight to nine years, difficulty in getting up from a squatting position for seven to eight years, nasal tone for seven to eight years, and breathlessness on climbing three to four flight of stairs and doing daily household activities for four to five years. The patient was alright nine years back when it was noticed by relatives and friends that the patient had some defect in walking, but the patient was unaware of it; however, after six to seven months, the patient presented to the private hospital for bilateral lower limb weakness and difficulty in walking and high-stepping gait as shown in the video. The weakness was insidious in onset and gradually progressive in nature. It gradually progressed in such a way that the patient could not do his daily household activities.

The patient also developed blurring of vision and on ophthalmological examination, was diagnosed with cataracts; the patient also had deranged blood sugar. The patient has a similar history to his mother and maternal uncle. On asking leading questions to the patient, it was also confirmed that he had good intellect and was studying law. The patient could not hold his neck in a lying down position and had difficulty swallowing. As the disease progressed, the patient was referred to a tertiary center for further management.

**Figure 1:**
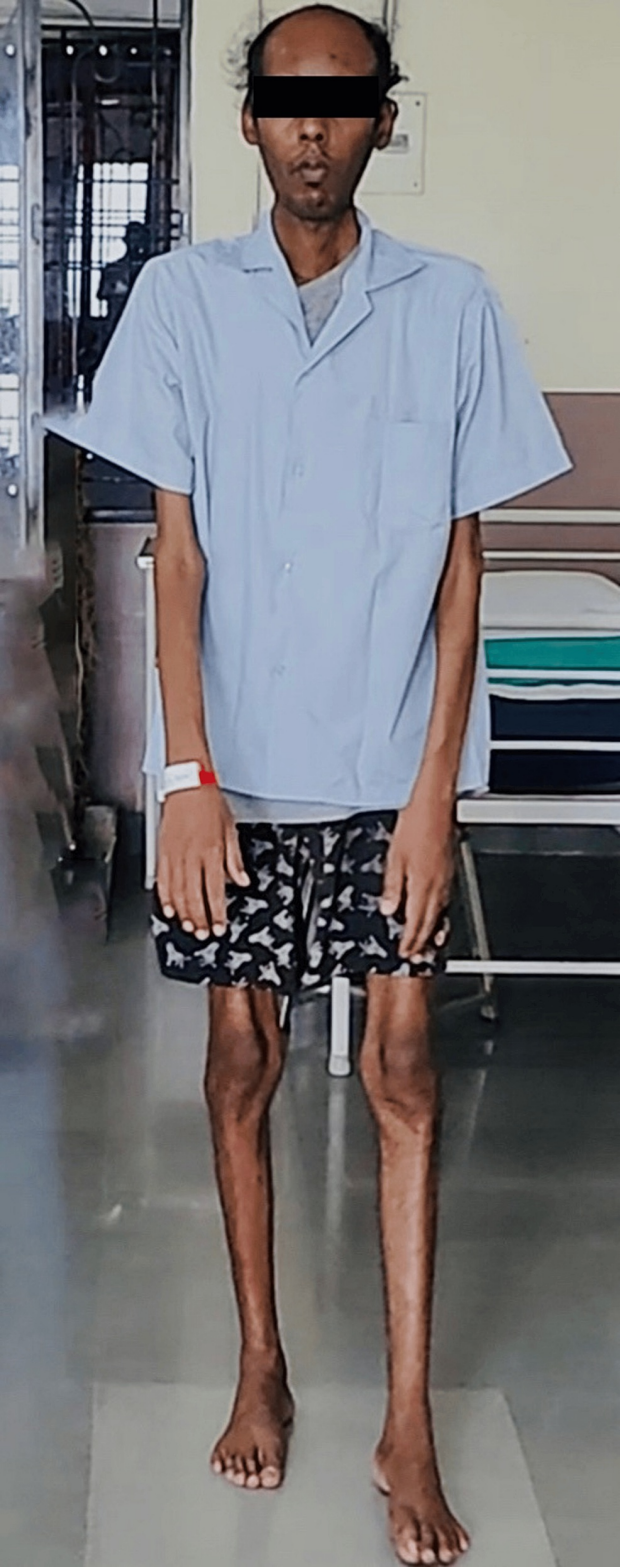
Severe wasting of bilateral lower limbs and upper limbs.

**Figure 2:**
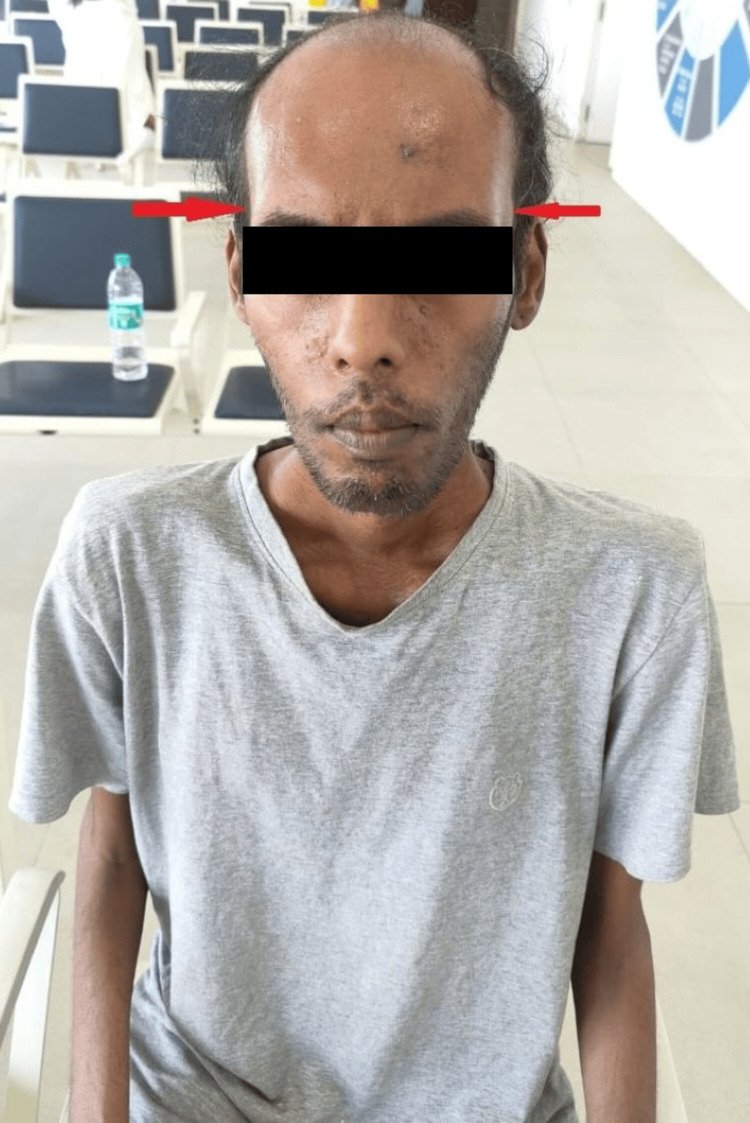
Temporal hallowing (emaciation) with a hatchet face and frontal balding (red arrow).

On general examination, the patient was thinly built, had a body mass index of 17 kg/m2, was afebrile, and had a pulse rate of 90 beats per minute, a blood pressure of 100/60 mmHg, a respiratory rate of 18 breaths per minute, and a SpO2 of 97% on room air. The patient was cooperative, conscious, and well-oriented to time, place, and person. The patient had severe wasting of bilateral lower limbs with temporal hollowing as well as frontal baldness as shown in Figures 1, 2, and Video 1. On further central nervous examination, all deep tendon reflexes were normal with no other focal neurological deficit. The cardiovascular and respiratory system examinations were normal. Reports of all routine investigations are highlighted in Table 1.

**Video 1:** High-stepping gait and severe wasting of muscles.

**Table 1:** Investigation profile of the patient.

Lab Parameters	Observed Value	Normal Range
Hemoglobin	14.2 gm%	13-17 gm%
Mean corpuscular volume	83.9 fL	83-101 fL
Total leucocyte count	5200 cells/ mm^3^	4000 – 10000 cells/ mm^3^
Platelets	239,000 mm^3^	150,000 – 400,000 mm^3^
Urea	29 mg/dL	19-43mg/dL
Creatinine	1.0 mg/dL	0.66-1.25mg/dL
Sodium	140 mmol/L	137-145 mmol/L
Potassium	4.1 mmol/L	3.5 – 5.1mmol/L
Alkaline phosphatase	124 U/L	38-126 U/L
Alanine transaminase	28 U/L	< 50 U/L
Aspartate aminotransferase	83 U/L	17-59U/L
Albumin	4.3 g/dL	3.5-5 g/dL
Total bilirubin	3.9 mg/dl	0.2-1.3mg/dl
Conjugated bilirubin	2.8 mg/dl	0.0-0.3 mg/dl
Unconjugated bilirubin	1.70 mg/dl	0.0-1.1 mg/dl
Testosterone	600 ng/dl	300-1000 ng/dl
Creatine phosphokinase	2061.4 IU/L	15-130 IU/L

His two-dimensional echocardiography (2D ECHO) revealed a left ventricular ejection fraction of 60% having normal valves. MRI brain with spinal screening was done and revealed no significant abnormality. Genetic tests for both dystrophia myotonica 1 (DM1) and dystrophia myotonica 2 (DM2) were done and were normal. His pulmonary function test showed severe obstruction with severe restriction with poor resolution post-bronchodilator. Both testicles were normal: right (length 20mm and breadth 22mm) and left (length 20mm and breadth 18mm in size), which were measured by ultrasonography.

The needle examination showed increased insertion activity with myotonic discharges waning and waxing spontaneously. Short-duration polyphasic motor unit action potentials (MUAPs) were predominant and an early recruitment pattern was seen in bilateral abductor pollicis brevis, deltoid, first dorsal interosseous, and mentalis muscles. On nerve conduction study, his distal latency compound muscle action potential (CMAP) amplitude was normal. He had normal conduction velocity in the median, ulnar nerve, peroneal nerve, and tibial nerve on both sides. Sensory nerve conduction showed normal sensory nerve action potential (SNAP) amplitude from the median nerve, ulnar nerve, and sural nerve on both sides. Electromyography (EMG) was done, which showed normal F wave latency from both lower limbs.

## Discussion

PROMM is reported as a multisystemic hereditary disease featuring proximal weakness, myotonia, tremors, cataracts, pain, cardiac disturbance, and hypogonadism due to such closed resemblance that it is generally confused with myotonic dystrophy. Both diseases are two different autosomal dominant disorders with separate gene loci. Chromosome 19 is the gene locus for myotonic dystrophy, whereas the gene locus for proximal myotonic myopathy is not known. On laboratory investigation, PROMM patients generally have elevated creatine kinase and liver enzyme (g-glutamyltransferase); some patients with PROMM may develop insulin resistance and some may show a disturbed cardiac rhythm and cardiomyopathy [4].

Myotonic dystrophy leads to shrinkage and weakness of muscle tissue and also affects the other organs in the body. Myotonic dystrophy typically has an autosomal dominant inheritance pattern. Further, myotonic dystrophies are divided into two types: DM1 and DM2. DM1 and DM2 have similar phenotypes and are differentiated based on muscles primarily affected (distal or proximal), congenital anomalies, type of muscle fibers involved, etc. [5].

In a case report by K Ricker et al., three families are described with the disorder prevalently inherited with proximal muscle weakness, myotonia, and cataracts [6]. The CTG expansion of the myotonic dystrophy gene is normal in the affected individuals, which is comparatively abnormal in myotonic dystrophy. Hence the gene study of the following subject explains no linkage of the myotonic dystrophy gene with myotonic dystrophy. The collection of symptoms in these three families seems to represent a new disorder [6,7].

As the nature of the disease is unknown, there is no specific treatment for the underlying disease process in PROMM. Only supportive treatments such as cataract extraction, rectification of cardiac arrhythmias, and pacemaker application if necessary as well as rehabilitation play a vital role in improving quality of life. Currently, genetic counseling is difficult in PROMM and there is limited data to check the severity of disease due to application. The risk of congenital PROMM is also not known. However, PROMM is comparatively milder than myotonic dystrophy, but few patients have reported premature death due to cardiac arrhythmias [8,9].

## Conclusions

PROMM should be considered in a patient who clinically suggests myotonic dystrophy but has a normal CTG test on the genetic study. Physicians should be aware that not every patient with PROMM has myotonia or cataracts. Genetic evaluation for myotonic dystrophy may turn out to be normal and some patients may only present with leg pain or mild proximal weakness, which needs to be vigilantly evaluated clinically and by a diagnostic method such as EMG, which may further lead to the diagnosis of PROMM, like in our case. Hence awareness of such scenarios where the genetic evaluation is normal but clinically significant signs and symptoms are present should direct the practitioner toward the diagnosis of PROMM. 

## References

[1] Nishihara N, Tachibana S, Sonoda H, Yamakage M (2020). A patient with myotonic dystrophy diagnosed after experiencing sudden respiratory failure: a case report. JA Clin Rep.

[2] Ehler E, Novotná A, Mareš M, Mušová Z, Mrklovský M (2012). Myotonic dystrophy type 2 and multiple sclerosis: case report. Clin Neurol Neurosurg.

[3] Jia YX, Dong CL, Xue JW, Duan XQ, Xu MY, Su XM, Li P (2022). Myotonic dystrophy type 1 presenting with dyspnea: a case report. World J Clin Cases.

[4] Moxley III RT, Udd B, Ricker K (1998). Proximal myotonic myopathy (PROMM) and other proximal myotonic syndromes. Neuromuscul Disord.

[5] Turner C, Hilton-Jones D (2010). The myotonic dystrophies: diagnosis and management. J Neurol Neurosurg Psychiatry.

[6] Ricker K, Koch MC, Lehmann-Horn F, Pongratz D, Otto M, Heine R, Moxley RT 3rd (1994). Proximal myotonic myopathy: a new dominant disorder with myotonia, muscle weakness, and cataracts. Neurology.

[7] Schneider C, Reiners K, Toyka KV (2001). Myotonic dystrophy (DM/Curschmann-Steinert disease) and proximal myotonic myopathy (PROMM/Ricker syndrome). Myotonic muscle diseases with multisystemic manifestations [Article in German]. Nervenarzt.

[8] Reddy V, Saboo K, Reddy K, Kumar S, Acharya S (2023). Pantothenate kinase-associated neurodegeneration (PKAN) with concomitant blepharospasm: unveiling a clinical enigma. Cureus.

[9] Gulrandhe P, Acharya S, Patel M, Shukla S, Kumar S (2023). Pertinence of constraint-induced movement therapy in neurological rehabilitation: a scoping review. Cureus.

